# Gut microbiota dysbiosis in ankylosing spondylitis: a systematic review and meta-analysis

**DOI:** 10.3389/fcimb.2024.1376525

**Published:** 2024-10-01

**Authors:** Qin-Yi Su, Yan Zhang, Dan Qiao, Xia Song, Yang Shi, Zhe Wang, Chen-Yan Wang, Sheng-Xiao Zhang

**Affiliations:** ^1^ Department of Rheumatology, The Second Hospital of Shanxi Medical University, Taiyuan, Shanxi, China; ^2^ Shanxi Provincial Key Laboratory of Rheumatism Immune Microecology, The Shanxi Medical University, Taiyuan, Shanxi, China; ^3^ Key Laboratory of Cellular Physiology at Shanxi Medical University, Ministry of Education, Taiyuan, China; ^4^ Shanxi Medical University (SXMU)-Tsinghua Collaborative Innovation Center for Frontier Medicine, Shanxi Medical University, Taiyuan, China

**Keywords:** ankylosing spondylitis, gut microbiota, dysbiosis, α-diversity, meta-analysis

## Abstract

**Background:**

Ankylosing spondylitis (AS) is a connective tissue disease that primarily affects spinal joints, peripheral joints, and paravertebral soft tissues, leading to joint stiffness and spinal deformity. Growing evidence has implicated gut microbiota in the regulation of AS, though the underlying mechanisms remain poorly understood.

**Methods:**

We conducted a comprehensive search of PubMed, Embase, Web of Science, the Cochrane Library, MEDLINE, Wanfang Data, China Science and Technology Journal Database (VIP), and China National Knowledge Internet (CNKI) databases from the time the databases were created until 30 July 2023. To evaluate changes in α-diversity and the abundance of certain microbiota families in AS, standardized mean difference (SMD) and 95% confidence interval (CI) calculations were made. Meta-analyses were performed using STATA 12.0 and the quality of the literature was assessed by following systematic review guidelines.

**Results:**

This systematic review and meta-analysis included 47 studies, providing insights into the gut microbiota composition in patients with AS compared to healthy controls (HCs). Our findings indicate a significant reduction in gut microbial diversity in patients with AS, as evidenced by a decrease in both richness and evenness. Specifically, the Shannon index showed a moderate decrease (SMD = -0.27, 95% CI: -0.49, -0.04; P < 0.001), suggesting a less diverse microbial ecosystem in patients with AS. The Chao1 index further confirmed this trend, with a larger effect size (SMD = -0.44, 95% CI: -0.80, -0.07; P < 0.001), indicating a lower species richness. The Simpson index also reflected a significant reduction in evenness (SMD = -0.30, 95% CI: -0.53, -0.06; P < 0.001). Additionally, patients with AS who received anti-rheumatic combination treatment exhibited a more pronounced reduction in α-diversity compared to untreated patients, highlighting the potential impact of this treatment on gut microbiota balance. In terms of specific microbial families, we observed a significant decrease in the abundance of Bifidobacterium (SMD = -0.42, 95% CI: -2.37, 1.52; P < 0.001), which is known for its beneficial effects on gut health. Conversely, an increase in the abundance of Bacteroidetes was noted (SMD = 0.42, 95% CI: -0.93, 1.76; P < 0.001), suggesting a possible shift in the gut microbiota composition that may be associated with AS pathophysiology.

**Conclusion:**

Our analysis revealed changes in α-diversity and the relative abundance of specific bacteria in AS. This suggests that targeting the gut microbiota could provide new therapeutic opportunities for treating AS.

**Systematic review registration:**

https://www.crd.york.ac.uk./PROSPERO/, identifier CRD42023450028.

## 1 Introduction

Ankylosing spondylitis (AS) is a chronic inflammatory rheumatic disease characterized by its primary impact on the axial skeleton, including the spine and sacroiliac joints, often leading to significant morbidity through joint stiffness and spinal deformity. The etiology of AS is multifactorial, with genetic, environmental, and immunological factors playing a role. Recent research has implicated the gut microbiota as a potential contributor to the pathogenesis of AS, suggesting a complex interplay between the intestinal microbiome and the host’s immune system (Asquith et al., 2019).

While the exact mechanisms by which the gut microbiota influences AS are not fully understood, studies have reported alterations in the composition and diversity of the gut microbiota in patients with AS. For instance, Zena Chen et al (Chen et al., 2021) and Hong Ki Min et al (Min et al., 2023) have identified specific changes in the abundance of Bacteroides and Faecalibacterium in patients with AS. These findings have sparked interest in the role of gut microbiota in AS, but the current literature presents a landscape of conflicting reports regarding the specific changes in gut microbiota abundance in patients with AS. Further, the impact of treatment on the gut microbiota of patients with AS is another area of active research. Medications such as disease-modifying anti-rheumatic drugs (DMARDs), nonsteroidal anti-inflammatory drugs (NSAIDs), and tumor necrosis factor (TNF) inhibitors are known to ameliorate symptoms and reduce inflammatory cytokine levels in patients with AS. Some studies have suggested that these treatments may also influence the gut microbiota composition (Chen et al., 2021), although the evidence is not yet conclusive.

Given the limited research on the alterations in gut microbiota associated with AS and the unclear underlying causes, there is a pressing need for a comprehensive synthesis of the current evidence. This study aims to systematically review the literature on the relationship between gut microbiota and AS, with a focus on understanding the nature of the association and the potential for intervention. This will contribute to the growing understanding of the role of the gut microbiota in AS and identify areas where further investigation is warranted.

## 2 Materials and methods

### 2.1 Data sources and search strategy

The association between AS and gut microbiota was assessed by searching the PubMed, Embase, Web of Science, the Cochrane Library, MEDLINE, Wanfang Data, VIP, and CNKI databases from their establishment to 30 July 2023. Key search terms include gastrointestinal microbiome, microbiome, gastrointestinal tract, intestinal microbiome, bacteria, intestinal tract, microflora, gastrointestinal tract, ankylosing spondylitis, axial spondyloarthritis, and their synonyms. The preferred reporting items for the systematic review and meta-analysis (PRISMA) report are followed by this study, which has been preregistered with PROSPERO (CRD42023450028).

### 2.2 Study selection and data extraction

All included studies performed research at the population level; the relationship between gut microbiota and AS was studied, the changes in gut microbiota indexes in patients with AS and healthy controls (HCs) were compared, and data for analysis were available.

Inclusion criteria:

1) Human research
2) Studies on the changes of gut microbiota in patients with AS.

Exclusion criteria:

1) Duplicate references in different databases.
2) Review articles, guidelines, conference abstracts, or case reports.
3) Incomplete data or unable to obtain full text.
4) No healthy control groups.
5) No available data were reported on intestinal flora α-diversity or intestinal microbial community composition.

We extracted the following data from the selected studies: essential information including first author, publication year, and country; clinical information including case numbers, genders, ages, α-diversity index in patients and healthy people, and the relative abundance of intestinal microorganisms; experimental data including fecal collection method, storage method, and DNA extraction technology.

### 2.3 Quality assessment

The quality assessment of included articles was evaluated using the Newcastle–Ottawa quality assessment scale (NOS). The NOS rates the risk of bias of case-control studies on the premises of appropriateness of sample frame, sampling method, ascertainment of exposure, a demonstration that the outcome of interest was not present at the start of the study, comparability of cohorts, methods for assessment of outcomes, duration of follow-up, and adequacy of follow-up.

### 2.4 Statistical analysis

The analysis was performed using Review Manager 5.4. Because the measured data of human intestinal flora are continuous variables, standardized mean difference (SMD) and its 95% confidence interval were used as the effect index to analyze the results. The results included in this analysis were tested for heterogeneity, and the heterogeneity among the results was judged according to the I² of the included results. When the results were homogenous (P > 0.1, I²≤50%), the fixed-effect model was used. In addition, we have analyzed using the random-effects model. When there was heterogeneity among study results (P ≤ 0.1, I² > 50%), the source of heterogeneity was analyzed, and subgroup analysis or sensitivity analysis was conducted for the studies. In this study, the outcome indexes with high heterogeneity were further analyzed by subgroup according to the specific type of IA, and sensitivity analysis was used to explore the sources of heterogeneity in the study.

## 3 Results

### 3.1 Study selection and characteristics

**Figure 1:**
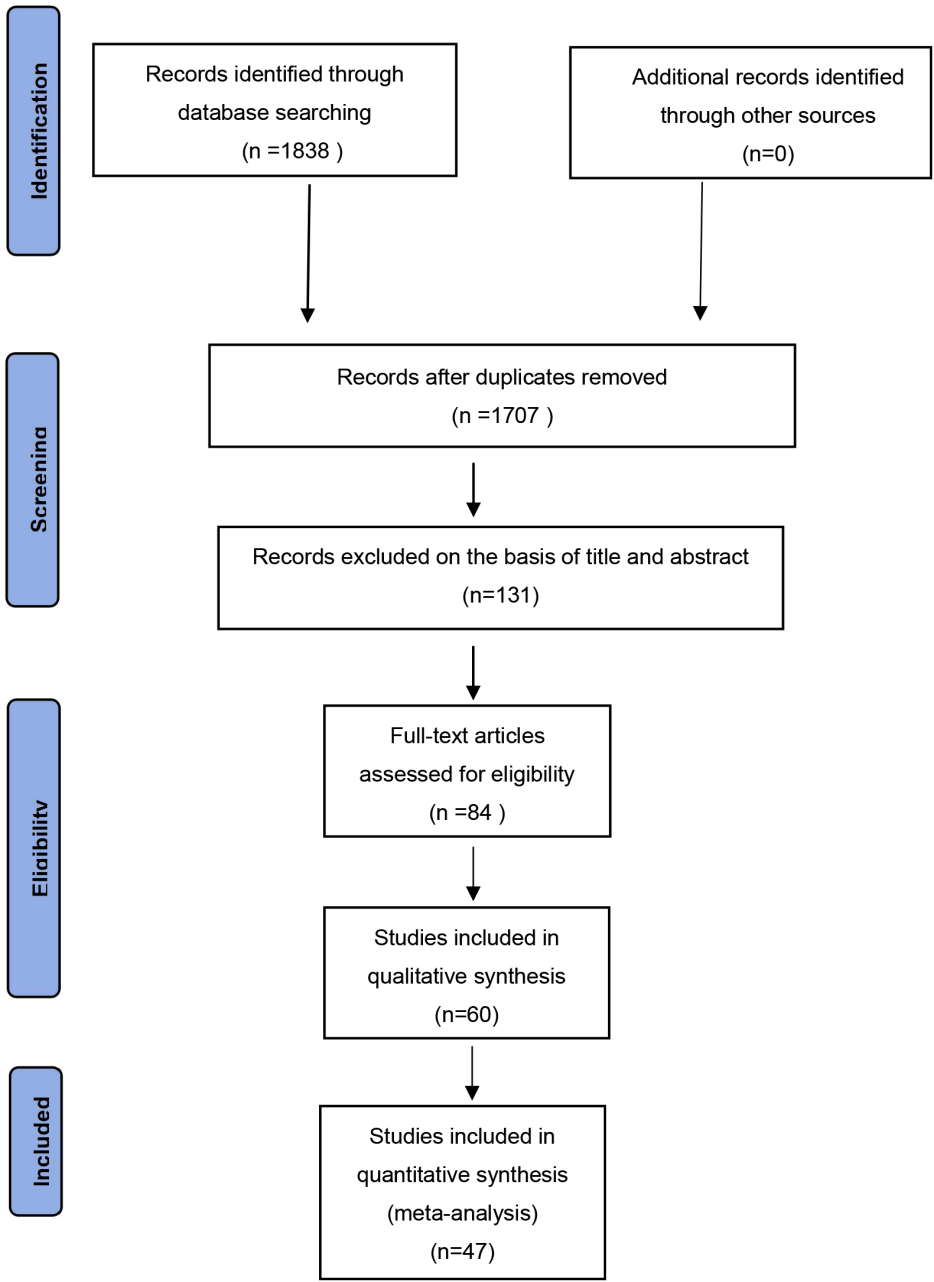
Retrieval flow chart. A total of 47 studies examined 2,494 patients with AS and 1,885 HCs. They all used human feces as the experimental samples and were observational case-control studies. According to the NOS, each included study was high quality (NOS score: 6-8) (**Table 1**).

Electronic database searches yielded 1,838 articles. After the title and abstract review, 1,707 articles were excluded because they did not meet the inclusion criteria. A total of 131 full-text articles were retrieved. After further screening for eligibility, 84 articles were excluded because they did not meet the eligibility criteria, and a total of 47 articles were eligible (**Figure 1**; **Table 1**).

**Table 1:** Basic information included in the study.

Study	Concomitant treatment	Q*	Technology employed	Platform
Chen Zhou-2018 (Zhou, 2018)	NA	7	16S rRNA gene sequencing	HiSeq2000
Xiutao Wang-2022 (Wang and Wang, 2022)	NA	7	16S rRNA gene sequencing	Illumina Miseq sequencing platform
Min, Hong Ki-2023 (Min et al., 2023)	NA	8	16S rRNA gene sequencing	Tapestation 4200
Sternes, P. R.-2022 (Sternes et al., 2022)	NA	9	16S rRNA gene sequencing	NA
Anca Cardoneanu -2021 (Cardoneanu et al., 2021)	TNF-α:67.85(19/28); SSZ:17.85(5/28); NSAIDs:14.28(4/28)	7	NA	NA
Chen Zhou-2020 (Zhou et al., 2020)	NA	7	16S rRNA gene sequencing	Illumina Miseq sequencing platform
Li Zhang -2019 (Zhang et al., 2019)	NSAIDs:68.9(71/103); biological agents:44.7(46/103); DMARDs:34.0(35/103)	7	16S rRNA gene sequencing	Illumina Miseq sequencing platform
Maxime Breban -2017 (Breban et al., 2017)	NSAIDs:51(25/49); Corticosteroids:12.2(6/49); DMARDs:4(2/49); Biotherapy:30.6(15/49); Antiacid:30.6(15/49)	6	16S rRNA gene sequencing	Illumina Miseq sequencing platform
Mary-Ellen Costello -2015 (Costello et al., 2015)	NSAIDs:11.1(1/9)	5	16S rRNA gene sequencing	NA
Xin Wang-2022 (Song et al., 2023)	NA	8	16S rRNA gene sequencing	NA
Sun, G.-2021 (Sun et al., 2021)	NA	5	16S rRNA gene sequencing	NA
Zhang, F.-2020 (Zhang et al., 2020a)	AbbVie:100(20/20)	6	16S rRNA gene sequencing	Illumina Miseq sequencing platform
Chen, Zena-2019 (Chen et al., 2019)	NA	9	16S rRNA gene sequencing	Illumina Miseq sequencing platform
Costello, M. E.-2015 (Costello et al., 2015)	NA	4	16S rRNA gene sequencing	NA
Li, M.-2019 (Li et al., 2019)	NSAIDs:40.91(9/22); BLs:36.36(8/22)	7	16S rRNA gene Sequencing	Illumina Miseq sequencing platform
Qinghong Dai-2022 (Dai et al., 2022)	NA	5	16S rRNA gene sequencing	Illumina Miseq sequencing platform
Bin Dou-2022 (Dou, 2022)	Antibody:29.2;Recombinant fusion protein:70.8;	4	16S rRNA gene sequencing	Illumina Miseq sequencing platform
Wen, C.-2017 (Wen et al., 2017)	NA	7	16S rRNA gene sequencing	Illumina Miseq sequencing platform
Ziyi Song-2022 (Song et al., 2023)	NSAIDs:38.7(24/62); steroid hormone:3.2(2/62);immunosuppressant:14.5(9/62);biological agents:16.1(10/62)	8	16S rRNA gene sequencing	Illumina Miseq sequencing platform
Zena Chen-2021 (Chen et al., 2021)	NSAIDs:38.7(24/62)	7	16S rRNA gene sequencing	Illumina Miseq sequencing platform
Magali Berland-2023 (Berland et al., 2023)	NSAIDs:100(30/30)	6	whole-metagenome shotgun sequencing	5500 SOLiD Wildfire
Qinghong Dai-2022 (Dai et al., 2022)	NSAIDs:53.5	4	16S rRNA gene sequencing	Illumina Miseq sequencing platform
Jian Yin-2020 (Yin et al., 2020)	Sulfasalazine:26.67(26/97); TNFi:33.33(32/97)	6	Shotgun metagenome sequencing	Illumina Miseq sequencing platform
Gang Liu-2020 (Liu et al., 2020)	TNFi:52.8(67/127)	4	16S rRNA gene sequencing	Illumina Miseq sequencing platform
H. K. Min-2023 (Min et al., 2023)	TNFi:51.5(17/33); NSAIDs:78.8(26/33)	6	16S rRNA gene sequencing	Illumina Miseq sequencing platform
Fangze Zhang-2020 (Zhang et al., 2020b)	NA	7	16S rRNA gene sequencing	Illumina Miseq sequencing platform
Guangming Jiang-2022 (Jiang, 2022)	NSAIDs:78(71/91); DMARDs:37.6(35/93)	9	16S rRNA gene sequencing	Illumina Miseq sequencing platform
Xin Wang-2022 (Xin et al., 2022)	NA	7	16S rRNA gene sequencing	Illumina Miseq sequencing platform
Chen, Z-2019 (Chen et al., 2019)	NA	5	16S rRNA gene sequencing	Illumina Miseq sequencing platform

*Quality (Q) of each study was based on the Newcastle–Ottawa quality scale.

NA refers to missing data collection.

### 3.2 α-diversity

#### 3.2.1 α-diversity changes in the gut microbiota of patients with AS

The 47 studies that investigated α-diversity differences between patients with AS and HCs had a total of 2,494 patients and 1,885 controls. The indices evaluated were richness (Chao1) and combined measures of richness and evenness (Shannon and Simpson indices). The Shannon, Simpson, and Chao1 indices were the most frequently reported metrics in the literature.

**Figure 2:**
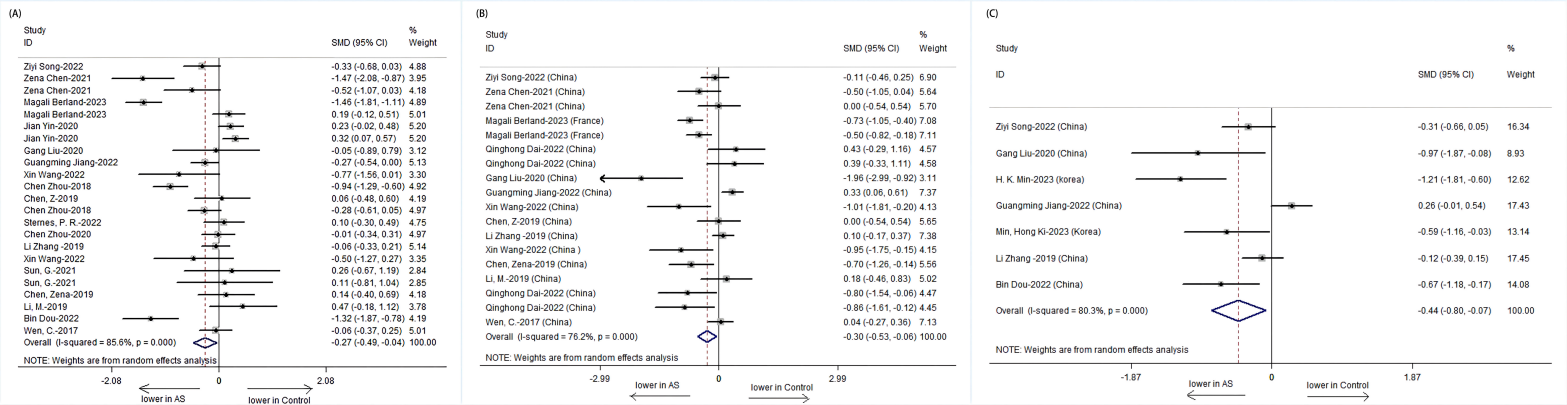
Forest plots of alterations in the α-diversity of patients with AS versus HCs: **(A)** Shannon index; **(B)** Simpson index; **(C)** Chao1.

The Chao1 index, indicative of species richness, was examined across seven studies and revealed a significant reduction in patients with AS compared to HCs (SMD = -0.44, 95% CI: -0.80 to -0.07; P < 0.001, heterogeneity I² = 80.3%) (**Figure 2C**). This suggests a lower microbial species count in the gut of AS patients.

The Shannon index, a measure of both richness and evenness, showed a significant decrease in 23 studies involving patients with AS (SMD = -0.27, 95% CI: -0.49 to -0.04; P < 0.001, I² = 85.6%) (**Figure 2A**). Similarly, the Simpson index, another metric of diversity, was significantly lower in 18 studies on patients with AS (SMD = -0.30, 95% CI: -0.53 to -0.06; P < 0.001, I² = 76.2%) (**Figure 2B**). These findings point toward an overall reduction in microbial diversity within the gut of patients with AS.

#### 3.2.2 Impact of treatment on α-diversity

**Figure 3:**
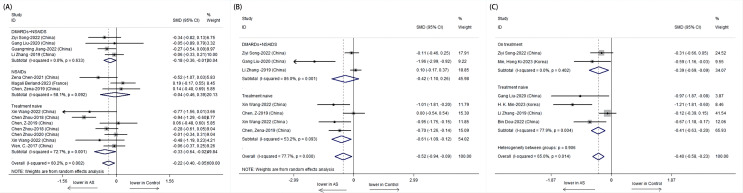
Forest plots of whether patients with AS receive treatment or not: **(A)** Shannon index. **(B)** Simpson index. **(C)** Chao1.

Further analysis stratified by treatment revealed distinct patterns in α-diversity indices. The Shannon index was notably lower in treatment-naive patients with AS compared to those receiving combined treatment with NSAIDs and DMARDs. The treatment-naive group showed a significant decrease in the Shannon index (SMD = -0.33, 95% CI: -0.64 to -0.02; P = 0.001), while the combined treatment group exhibited an increase in the Shannon index, suggesting a potential recovery of microbial diversity post-treatment (**Figure 3**).

**Figure 4:**
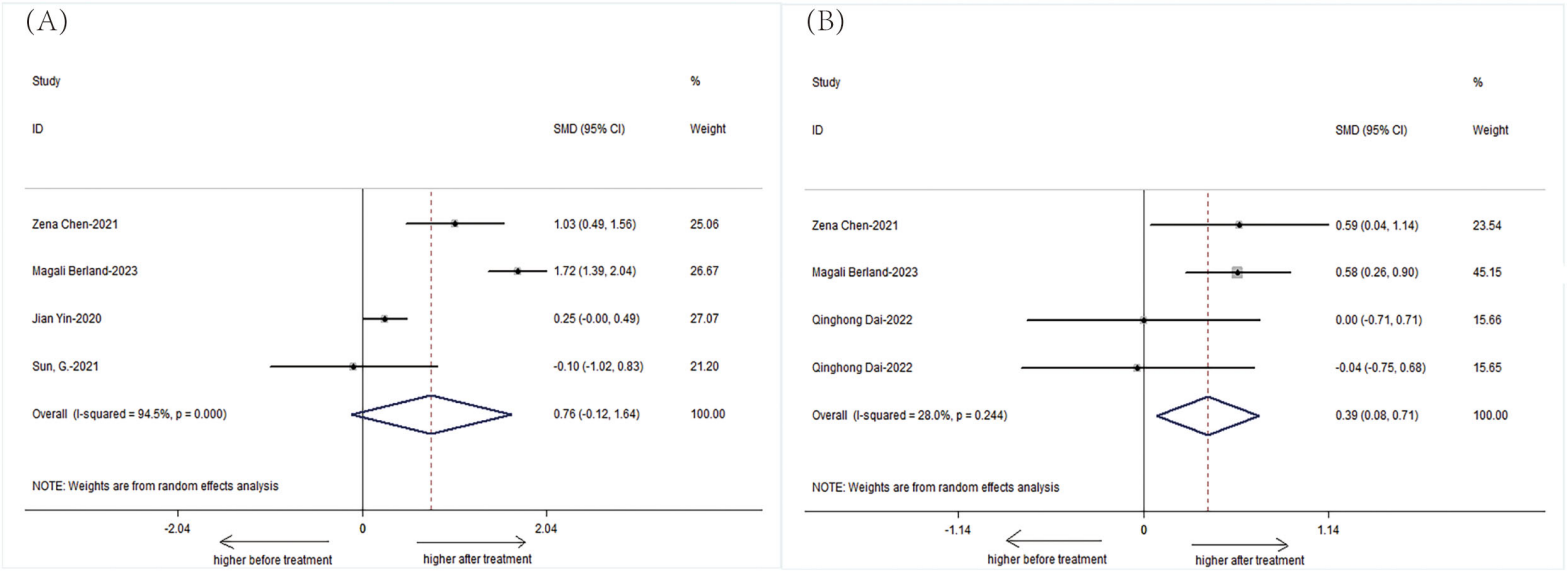
Forest plots of patients with AS before treatment and after treatment: **(A)** Shannon index. **(B)** Simpson index.

The Simpson index also depicted a significant difference among untreated AS patients, with a lower index indicating reduced diversity. However, no significant difference was observed in the combined treatment group, which may imply a stabilization or improvement in microbial evenness with treatment (**Figure 4**).

Subgroup analysis based on treatment type revealed that while the treatment-naive group had a significantly lower Shannon index, the combined treatment group showed a non-significant increase, suggesting a possible positive effect of combined therapy on microbial diversity. For the Simpson index, a significant difference was observed only in the untreated group, with the combined treatment group showing no significant change, indicating a potential normalization of microbial evenness with treatment.

**Figure 5:**
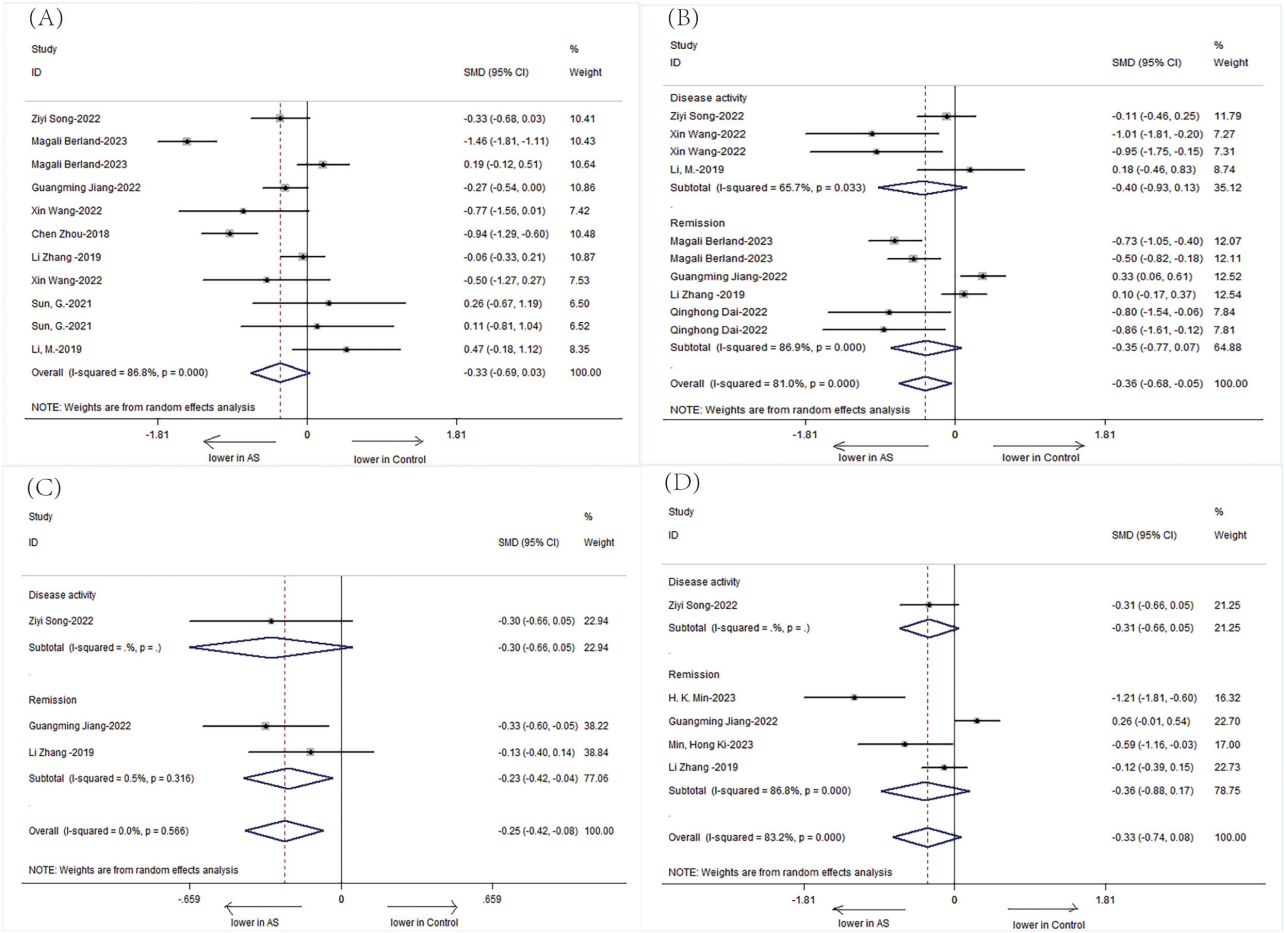
Forest plots of the disease activity of patients with AS: **(A)** Shannon index. **(B)** Simpson index. **(C)** ACE. **(D)** Chao1.

Additionally, we performed a subgroup analysis in 582 patients, stratified according to baseline disease activity. Patients were divided into two subgroups according to their disease activity as follows: inactive group and active group. There were significantly detected in AS patients in active group compared to AS patients in inactive group. (**Figure 5**: Shannon: SMD = -0.33; 95%CI: -0.69 to 0.03; P < Q19 0.001; Simpson: SMD = -0.36; 95%CI: -0.68 to -0.05; P < 0.001; ACE: SMD = -0.25; 95%CI: -0.42 to -0.08; P > 0.05; Chao1: SMD = -0.33, 95%CI: -0.74 to 0.08; P < 0.001).

#### 3.2.3 Changes in specific gut microbes in patients with AS

**Figure 6:**
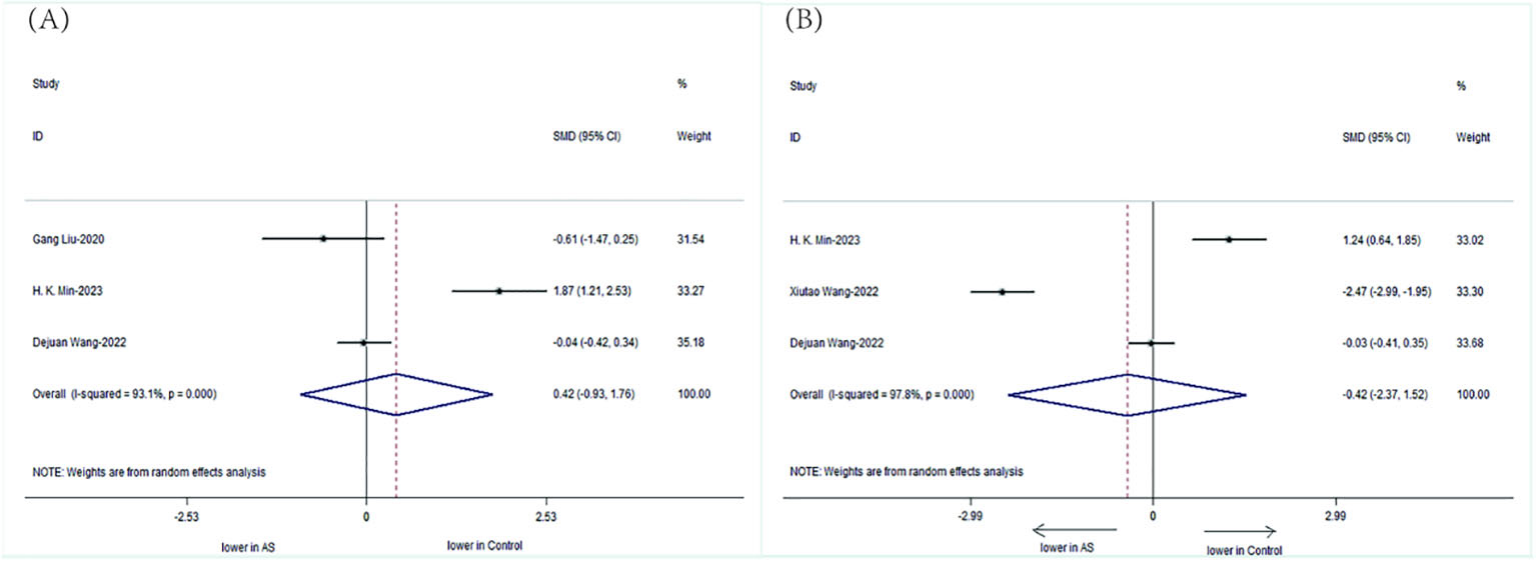
Forest plots of the alterations in the gut microbiota of patients with AS versus HCs: **(A)** *Bacteroidetes*, **(B)** *Bifidobacterium*.

We specifically analyzed the composition of intestinal microbial communities in patients with AS. The results showed that the patients with AS had a decreased abundance of *Bifidobacterium* (SMD=-0.42, 95%CI-2.37, 1.52;P < 0.001) but an increased abundance of *Bacteroidetes* (SMD= 0.42, 95%CI -0.93, 1.76; P < 0.001) compared with those of HCs (**Figure 6**).

#### 3.2.4 Analysis of publication bias and sensitivity

We used Egger’s, Begg’s, and funnel plots to test and assess publication bias risk (see the appendix in the electronic **Supplementary Material**). The Shannon index (t=-1.14, p=0.266), Simpson index (t=-1.42, p=0.174), Abundance-based Coverage Estimators (ACE) (t=1.33, p=0.253), and InvSimpson (t=-3.42, p=0.181) results showed no evidence of publication bias in included studies of indicating that the conclusions of the meta-analysis were relatively reliable. However, Egger’s test showed evidence of publication bias in the included studies in the Chao1 index (t =-4.46, p = 0.003). A sensitivity analysis examined whether the Shannon index, Simpson index, ACE, Chao1, InvSimpson, *Bacteroidetes*, or *Bifidobacterium* results were influenced by individual studies. There was no significant influence on the pooled results when any individual study was removed.

## 4 Discussion

The role of gut microbiota in the pathogenesis of AS has been a subject of considerable debate, with previous studies reporting conflicting findings regarding its diversity. This systematic review and meta-analysis aimed to clarify these discrepancies and assess the impact of treatment on the α-diversity of gut microbiota in patients with AS. In our study, we observed significant differences in α-diversity between patients with AS and HCs.

Our findings align with the growing body of evidence suggesting that patients with AS exhibit reduced α-diversity compared to HCs, indicating a potential dysbiosis that may contribute to the disease’s inflammatory processes. The observed differences in gut microbiota composition, particularly the notable changes in Bacteroidetes and Bifidobacterium, underscore the need for a deeper understanding of their immunomodulatory roles and their potential as therapeutic targets (Fan, 2021). The heterogeneity in the study results may be attributed to various factors, including differences in dietary habits, genetic predispositions, and geographical variations, which could influence the gut microbiota composition (Sun et al., 2019). The predominance of Chinese populations in our reviewed studies might reflect these dietary and lifestyle factors, highlighting the importance of considering such variables in future research. While our analysis did not reveal significant differences in other gut microbiota between patients with AS and HCs, it is essential to recognize that the gut microbiota’s complex ecosystem involves not only bacteria but also fungi, such as Candida albicans. The interplay between the gut fungal community and the host’s immune system is critical for maintaining homeostasis (Gutierrez and Arrieta, 2021; Lau et al., 2021).

In addition, C-reactive protein (CRP) levels, a marker of inflammation, have been shown to differ significantly between patients with AS and healthy individuals (Cao et al., 2015). The binding of CRP to bacterial components, such as choline phosphate, triggers an inflammatory response, suggesting a potential regulatory role of CRP in the gut microbiota-immune system interaction (Tillett and Francis, 1930; Scher et al., 2013). This warrants further investigation into the microbial regulatory properties of CRP.

Our study also examined the relationship between pharmacological treatments, such as NSAIDs and DMARDs, and the gut microbiota. While the effects of these treatments on the microbiota are not well documented, there is evidence to suggest that TNF inhibitors may restore gut microbiota balance (Chen et al., 2021; Ditto et al., 2021). The Simpson index findings in our study, showing lower diversity in patients with AS, may be influenced by various factors including detection methods, race, and diet. In addition, our study found that some patients had gastrointestinal complications, which may be related to drug therapy.

It is well-established that gut microbiota α-diversity is associated with disease activity in patients with AS (Wang et al., 2022). Our subgroup analysis supports this association and further implicates the HLA-B27 gene in the dysregulation of gut microbiota. The increased expression of the HLA-B27 antigen in target tissues may trigger an inflammatory cascade, suggesting a genetic component in AS pathogenesis (Cauli et al., 2012). Furthermore, in genetically susceptible individuals, such as patients with AS who are carriers of the HLA-B27 gene, increased intake of starchy foods may contribute to the development and development of AS or spondylitis-associated Crohn’s disease (CD). The control of diet to reduce the risk of disease is expected to become a new research direction.

Our meta-analysis, while comprehensive, has several limitations. The modest sample size of included studies may limit the power of our findings, necessitating further studies with larger cohorts. Additionally, the inclusion of articles only published in English and Chinese may introduce publication bias. The use of different computational pipelines for microbiota analysis could also affect the comparability of results. Finally, the lack of documentation on the effects of many drugs on the gut microbiota limits our ability to analyze their impact fully.

In summary, the gut microbiota’s role in AS is multifaceted, involving both direct and indirect effects on the host’s immune system and inflammatory pathways. Further research is needed to fully understand the mechanisms by which the gut microbiota influences AS and to develop targeted interventions that leverage the microbiota for the benefit of patients with this debilitating disease.

## 5 Conclusion

In summary, the findings presented in this thesis add to our understanding of the relationship between gut microbiota and AS. The α-diversity of patients with AS was decreased compared to HCs, and patients with AS had a decreased abundance of *Bifidobacterium* but an increased abundance of *Bacteroidetes*. In addition, we found that NSAIDs exhibit significant efficacy in treating AS. We hope that further tests will prove our theory.

## Data Availability

The original contributions presented in the study are included in the article/**Supplementary Material**. Further inquiries can be directed to the corresponding author.
