# Supplementary material for: Gut microbiota dysbiosis in ankylosing spondylitis: a systematic review and meta-analysis
Source: Front Cell Infect Microbiol. 2024 Oct 1;14:1376525. doi: 10.3389/fcimb.2024.1376525 (PMC11484232; doi:10.3389/fcimb.2024.1376525)
Supplement: Supplementary file 1 [file Table1.docx]

Supplementary Material

# Supplementary Table

**Table 1**: Basic information included in the study

| **Study** | **Concomitant treatment** | **Q*** | **Technology employed** | **Platform** |
| --- | --- | --- | --- | --- |
| Chen Zhou-2018[5] | NA | 7 | 16S rRNA gene  sequencing | HiSeq2000 |
| Xiutao Wang-2022[6] | NA | 7 | 16S rRNA gene  sequencing | Illumina Miseq sequencing platform |
| Min, Hong Ki-2023[7] | NA | 8 | 16S rRNA gene  sequencing | Tapestation 4200 |
| Sternes, P. R.-2022[8] | NA | 9 | 16S rRNA gene  sequencing | NA |
| Anca Cardoneanu -2021[9] | TNF-α:67.85(19/28); SSZ:17.85(5/28); NSAIDs:14.28(4/28) | 7 | NA | NA |
| Chen Zhou-2020[10] | NA | 7 | 16S rRNA gene  sequencing | Illumina Miseq sequencing platform |
| Li Zhang -2019[11] | NSAIDs:68.9(71/103); biological agents:44.7(46/103); DMARDs:34.0(35/103) | 7 | 16S rRNA gene  sequencing | Illumina Miseq sequencing platform |
| Maxime Breban -2017[12] | NSAIDs:51(25/49); Corticosteroids:12.2(6/49); DMARDs:4(2/49); Biotherapy:30.6(15/49); Antiacid:30.6(15/49) | 6 | 16S rRNA gene  sequencing | Illumina Miseq sequencing platform |
| Mary-Ellen Costello -2015[13] | NSAIDs:11.1(1/9) | 5 | 16S rRNA gene  sequencing | NA |
| Xin Wang-2022[14] | NA | 8 | 16S rRNA gene  sequencing | NA |
| Sun, G.-2021[15] | NA | 5 | 16S rRNA gene  sequencing | NA |
| Zhang, F.-2020[16] | AbbVie:100(20/20) | 6 | 16S rRNA gene  sequencing | Illumina Miseq sequencing platform |
| Chen, Zena-2019[17] | NA | 9 | 16S rRNA gene  sequencing | Illumina Miseq sequencing platform |
| Costello, M. E.-2015[18] | NA | 4 | 16S rRNA gene  sequencing | NA |
| Li, M.-2019[19] | NSAIDs:40.91(9/22); BLs:36.36(8/22) | 7 | 16S rRNA gene  Sequencing | Illumina Miseq sequencing platform |
| Qinghong Dai-2022[20] | NA | 5 | 16S rRNA gene  sequencing | Illumina Miseq sequencing platform |
| Bin Dou-2022[21] | Antibody：29.2；  Recombinant fusion protein：70.8； | 4 | 16S rRNA gene  sequencing | Illumina Miseq sequencing platform |
| Wen, C.-2017[22] | NA | 7 | 16S rRNA gene  sequencing | Illumina Miseq sequencing platform |
| Ziyi Song-2022[23] | NSAIDs:38.7(24/62);  steroid hormone:3.2(2/62);  immunosuppressant:14.5(9/62);  biological agents:16.1(10/62) | 8 | 16S rRNA gene  sequencing | Illumina Miseq sequencing platform |
| Zena Chen-2021[24] | NSAIDs:38.7(24/62) | 7 | 16S rRNA gene  sequencing | Illumina Miseq sequencing platform |
| Magali Berland-2023[25] | NSAIDs:100(30/30) | 6 | whole-metagenome shotgun sequencing | 5500 SOLiD Wildfire |
| Qinghong Dai-2022[26] | NSAIDs:53.5 | 4 | 16S rRNA gene  sequencing | Illumina Miseq sequencing platform |
| Jian Yin-2020[27] | Sulfasalazine:26.67(26/97); TNFi:33.33(32/97) | 6 | Shotgun metagenome sequencing | Illumina Miseq sequencing platform |
| Gang Liu-2020[28] | TNFi:52.8(67/127) | 4 | 16S rRNA gene  sequencing | Illumina Miseq sequencing platform |
| H. K. Min-2023[29] | TNFi：51.5(17/33); NSAIDs:78.8(26/33) | 6 | 16S rRNA gene  sequencing | Illumina Miseq sequencing platform |
| [Fangze Zhang-2020](https://pubmed.ncbi.nlm.nih.gov/?sort=date&term=Zhang+F&cauthor_id=32161018)[30] | NA | 7 | 16S rRNA gene  sequencing | Illumina Miseq sequencing platform |
| Guangming Jiang-2022[31] | NSAIDs:78(71/91); DMARDs:37.6(35/93) | 9 | 16S rRNA gene  sequencing | Illumina Miseq sequencing platform |
| Xin Wang-2022[32] | NA | 7 | 16S rRNA gene  sequencing | Illumina Miseq sequencing platform |
| Chen, Z-2019[33] | NA | 5 | 16S rRNA gene  sequencing | Illumina Miseq sequencing platform |

*Quality (Q) of each study was based on the Newcastle-Ottawa Quality.

**Table 2**: Basic information included in the study

| Study | Country | Sample size | Mean age(yr) | Female(%) | HLA B27 | *BASDAI* | *BASMI* | *CRP* |
| --- | --- | --- | --- | --- | --- | --- | --- | --- |
| Chen Zhou-2018 | China | P:85 | P:30.72 | P:21.2 | NA | NA | NA | NA |
|  |  | C:63 | C:33.68 | C:28.6 | NA | NA | NA | NA |
| Xiutao Wang-2022 | China | P:30 | P:40.17± 3.34 | P:23.30 | NA | NA | NA | 16.32± 3.81 |
|  |  | C:30 | C:40.52 ± 2.98 | C:26.70 | NA | NA | NA | 3.26 ± 0.83 |
| Min, Hong Ki-2023 | Korea | P:33 | P:42.3 ± 12.4 | P:9.1 | 32 (97.0%) | 2.9 ± 2.1 | NA | 0.3 ± 0.6 |
|  |  | C:20 | C:33.0 ± 5.7 | C:80 | NA | NA | NA | NA |
| Sternes, P. R.-2022 | Australia | P:33 | P:48.5 ± 15.3 | P:40 | 60 (95.2%) | NA | NA | NA |
|  |  | C:105 | C:62.2 ± 10.3 | C:46 | NA | NA | NA | NA |
| Anca Cardoneanu -2021 | Romania. | P: 28 | 52.1 ±13,6 | P:39.1 | NA | 4.83 | NA | NA |
|  |  | C: 32 | 61.5 ±10 | C:62.5 | NA | NA | NA | NA |
| Chen Zhou-2020 | China | P:85 | NA | NA | NA | NA | NA | NA |
|  |  | C:62 |  |  | NA | NA | NA | NA |
| Li Zhang -2019 | China | P:103 | 33.29 ± 11.66 | 17.50 | 95 (92.6%) | 3.06±2.07 | NA | 9.2(3.8,24.0) |
|  |  | C:105 | 33.66 ± 12.53 | 19.00 | NA | NA | NA | NA |
| Maxime Breban -2017 | France | P:49 | 48±13 | 55 | 67% | NA | NA | NA |
|  |  | C:18 | 37±10.4 | 50 | NA | NA | NA | NA |
| Mary-Ellen Costello -2015 | Australia | P:9 | P:34.4±9.95 | P:22.2 | 9（100%） | 6.14±0.96 | NA | 4.02±5.38 |
|  |  | C:9 | C :47.89±9.99 | C:66.7 | NA | NA | NA | 0.34±0.33 |
| Xin Wang-2022 | China | P:20 | 17-59 | P:10 | 20(66.7%) | NA | NA | >2.1 |
|  |  | C:10 | 17-60 | C:10 | NA | NA | NA |  |
| Sun, G.-2021 | China | P:9 | P:41.89 ± 7.29 | P:11.1 | 9（50%） | 6.40±0.25 | NA | NA |
|  |  | C:9 | C:47.56 ± 10.18 | C:66.7 | NA | NA | NA | NA |
| Zhang, F.-2020 | China | P:20 | P:33.18 ± 4.23 | P:0 | 20(100%) | ≥ 4 | 6.94 ± 1.36 | 4.01 ± 0.68 |
|  |  | C:19 | C:33.62 ± 3.35 | C:0 |  | NA |  |  |
| Chen, Zena-2019 | China | P: 41 | P: 29.90±10.03 | P: 15 | 40(97.56%) | NA | NA | 7.5±17.5； |
|  |  | C: 19 | C: 30.89±10.61 | C: 31.6 | NA | NA | NA | NA |
| Li, M.-2019 | China | P:22 | P:34.86(15–58) | P:0 | 19（85.71%） | 5.05(2.1–9.4) | NA | 14.52(0.1–67) |
|  |  | C:16 | C:34.35 | C:0 | NA | NA | NA | NA |
| Qinghong Dai-2022 | China | P:24 | P:32.3 ± 10.5 | P:8.3 | NA | Pretreatment：3.86 ± 0.78；Post-treatment：1.56 ± 0.68； | NA | Pretreatment：31.0 ± 18.6；Post-treatment：6.8 ± 11.8； |
|  |  | C:11 | C:35.1 ± 11.1 | C:18.2 | NA | NA | NA | NA |
| Bin Dou-2022 | China | P:34 | NA | NA | 34(100%) | NA | NA | NA |
|  |  | C:30 | NA | NA | 15(50%) | NA | NA | NA |
| Wen, C.-2017 | China | P:73 | 36.00 ±10 | 45.20 | 6（90.41%） | 3.32±2.19 | NA | 12.66±19.79 |
|  |  | C:83 | 41.96±8.52 | 42.17 | NA | NA | NA | ＜5 |
| Ziyi Song-2022 | China | P: 62 | P: 42.0±13.7 | P: 32.3 | 52（83.87%） | NA | NA | 15.9(5.2,35.9) |
|  |  | C: 62 | C: 42.0±13.5 | C: 32.3 | NA | NA | NA | NA |
| Zena Chen-2021 | China | P:30 | P: 31.23 ± 7.48 | P: 10 | 30（100%） | 30 | 2.50 (3.00) | 11.25 (21.70) |
|  |  | C: 24 | C: 38.54 ± 10.79 | NA | NA | NA | NA | NA |
| Magali Berland-2023 | France | P:102 | P:50±11.3 | P: 51 | 84（82.5%） | 3.78 ± 2.29 | NA | NA |
|  |  | C: 63 | C:48.7±11.7 | C: 60.3 | 27（43.1%） | NA | NA | NA |
| Jian Yin-2020 | China | P: 127 | NA | NA | NA | NA | NA | NA |
|  |  | C:123 | NA | NA | NA | NA | NA | NA |
| Gang Liu-2020 | China | P: 10 | P: 49.2±5.98 | 4(40%) | 13（100%） | NA | NA | 24.69±9.24 |
|  |  | C:12 | C:45.67±5.38 | 6(50%) | 0 | NA | NA | 1.57±0.86 |
| [Fangze Zhang-2020](https://pubmed.ncbi.nlm.nih.gov/?sort=date&term=Zhang+F&cauthor_id=32161018) | China | p:20 | p:33.18±4.23 | 0 | 20（100%） | 7 | NA | NA |
|  |  | c:19 | c:34.02±3.49 | 0 | NA | NA | NA | NA |
| Guangming Jiang-2022 | China | P:103 | P:33.29 ±11.66 | P:17.5 | NA | NA | NA | NA |
|  |  | C：104 | C:33.66±12.53 | C:19.2 | NA | NA | NA | NA |

## Supplementary Figures


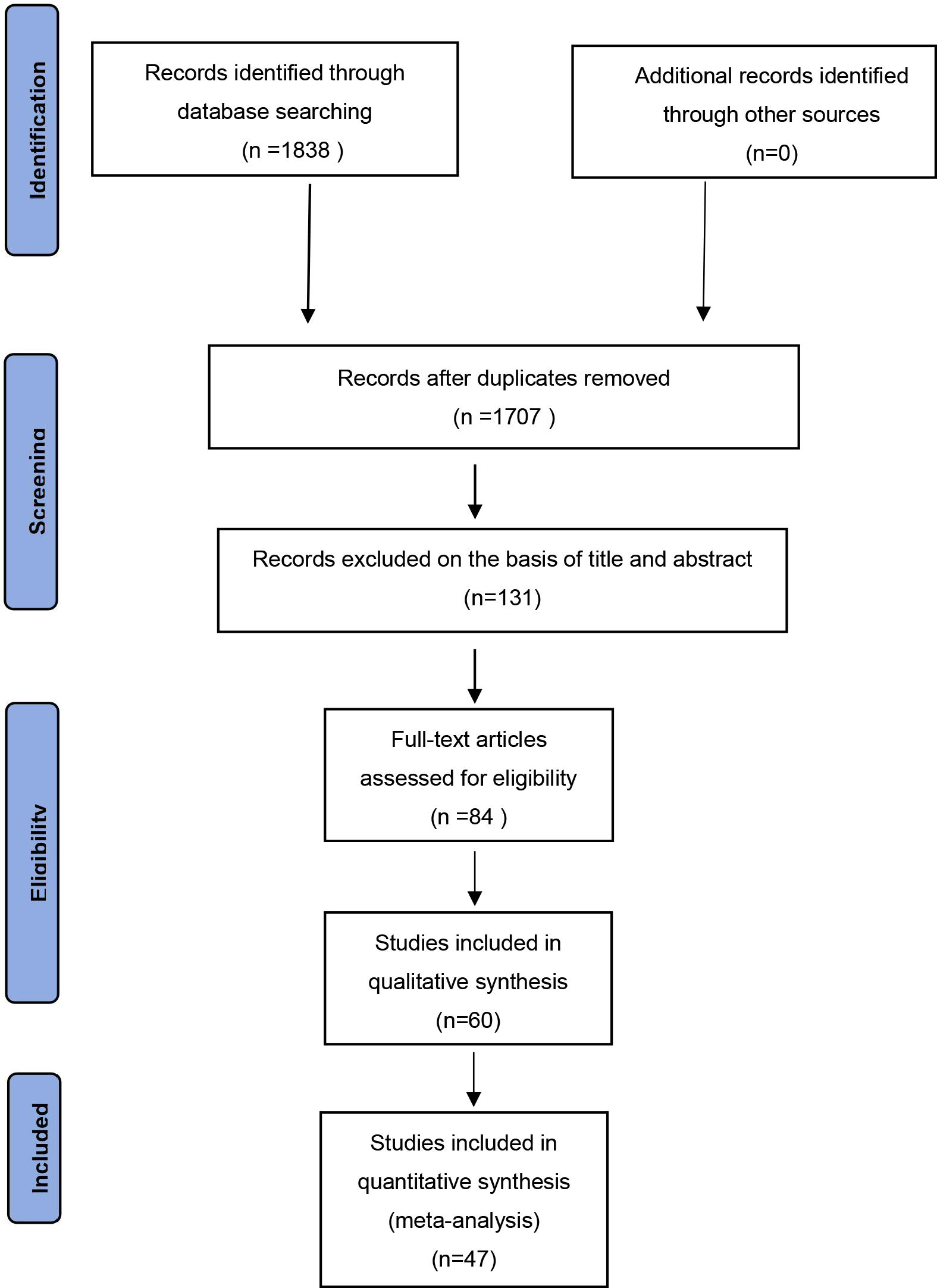


**Supplementary Figure 1.** Retrieval flow chart.


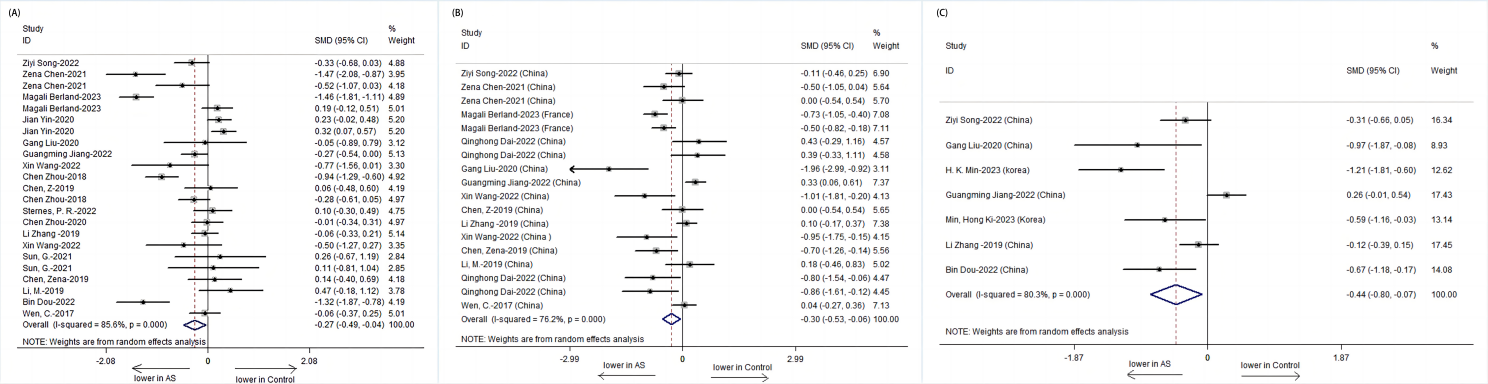


**Supplementary Figure 2.** Forest plots of alterations in the α-diversity of patients with AS versus HCs: (A) Shannon index; (B) Simpson index; (C) ACE; (D) Chao1.


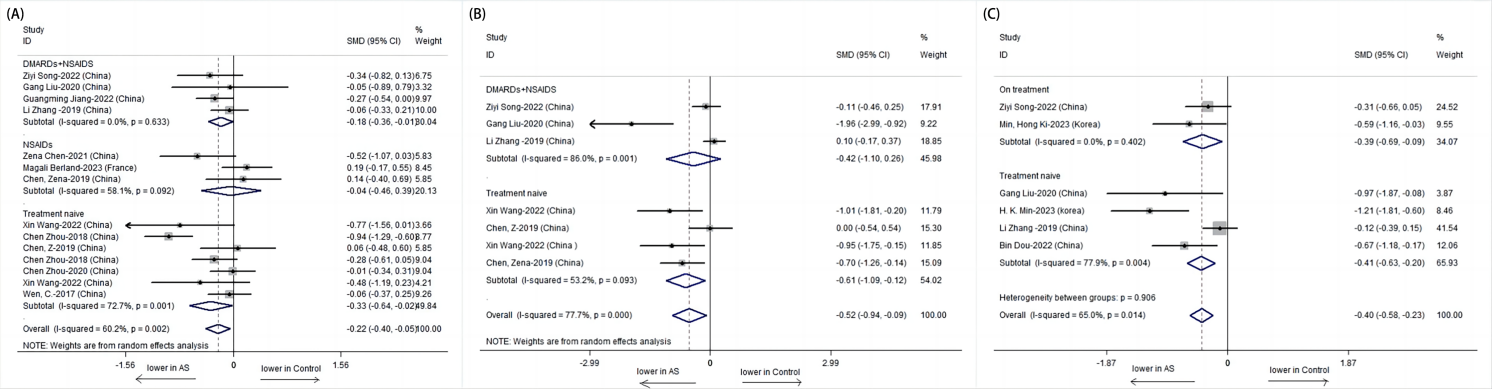


**Supplementary Figure 3.** Forest plots of whether patients with AS receive treatment or not: (A) Shannon index. (B) Simpson index. (C) ACE. (D) Chao1.


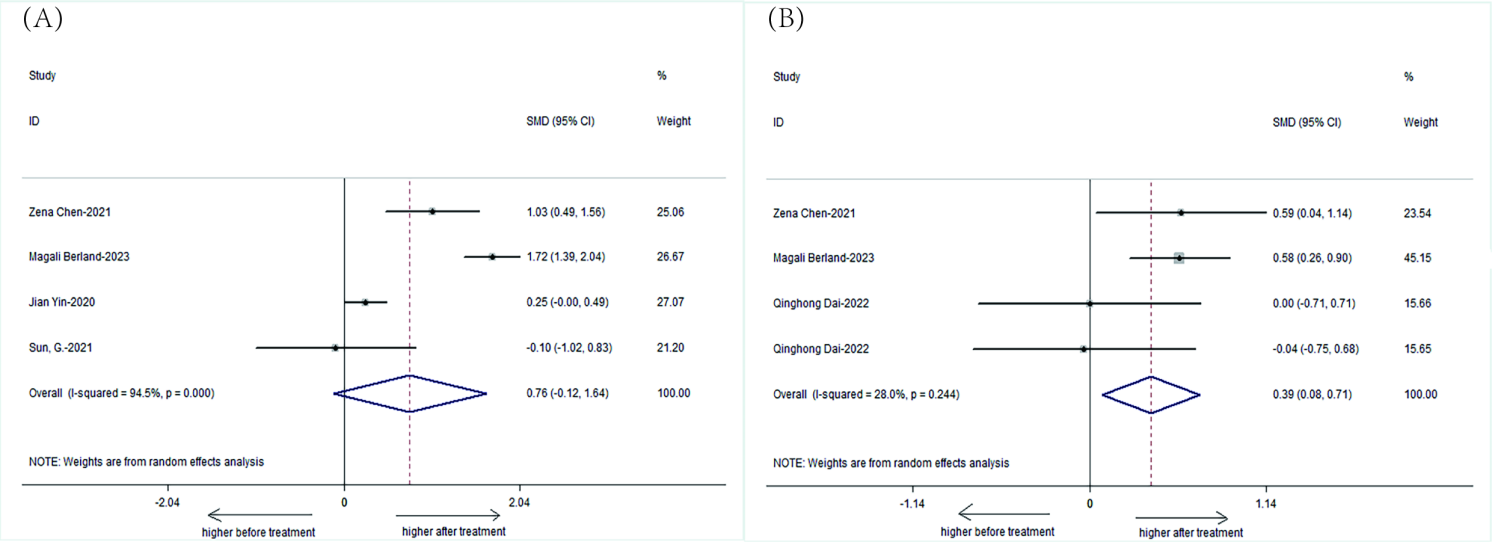


**Supplementary Figure 4.** Forest plots of patients with AS before treatment and after treatment : (A) Shannon index. (B) Simpson index.

##
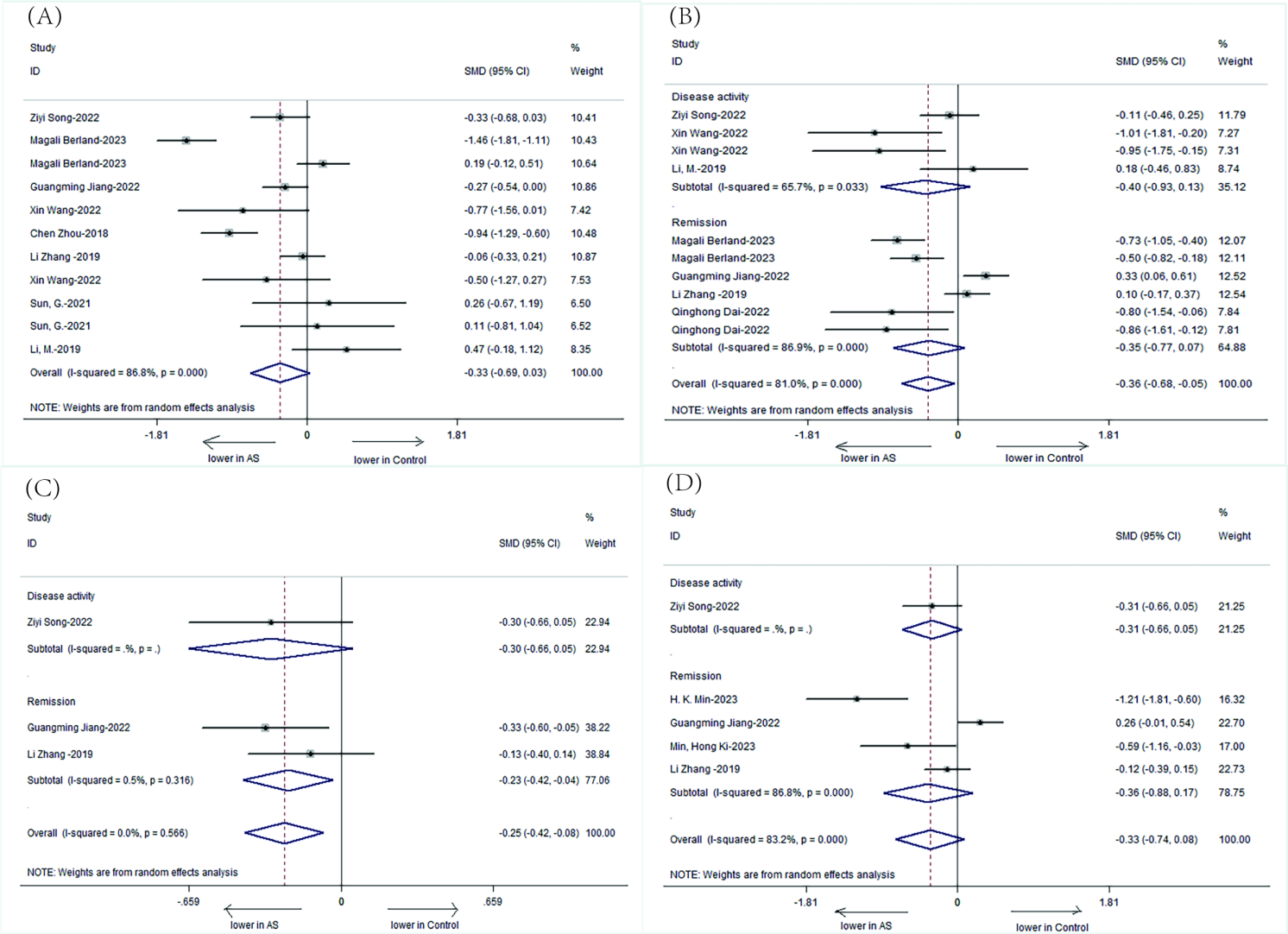


**Supplementary Figure 5.** Forest plots of the disease activity of patients with AS: (A) Shannon index. (B) Simpson index. (C) ACE. (D) Chao1.


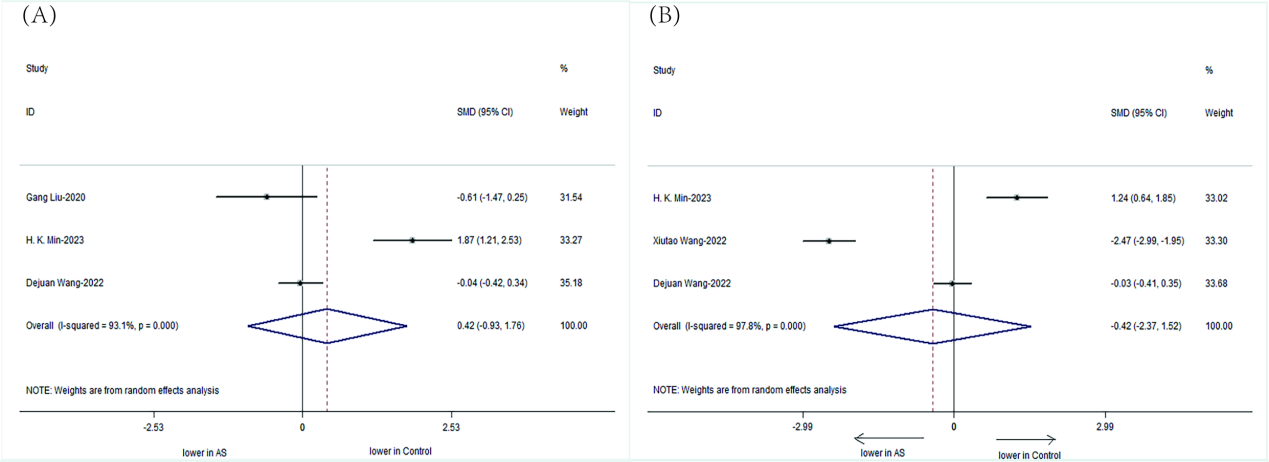


**Supplementary Figure 6.**Forest plots of alterations in the gut microbiota of patients with AS versus HCs:(A)*Bacteroidetes* (B) *Bifidobacterium*.


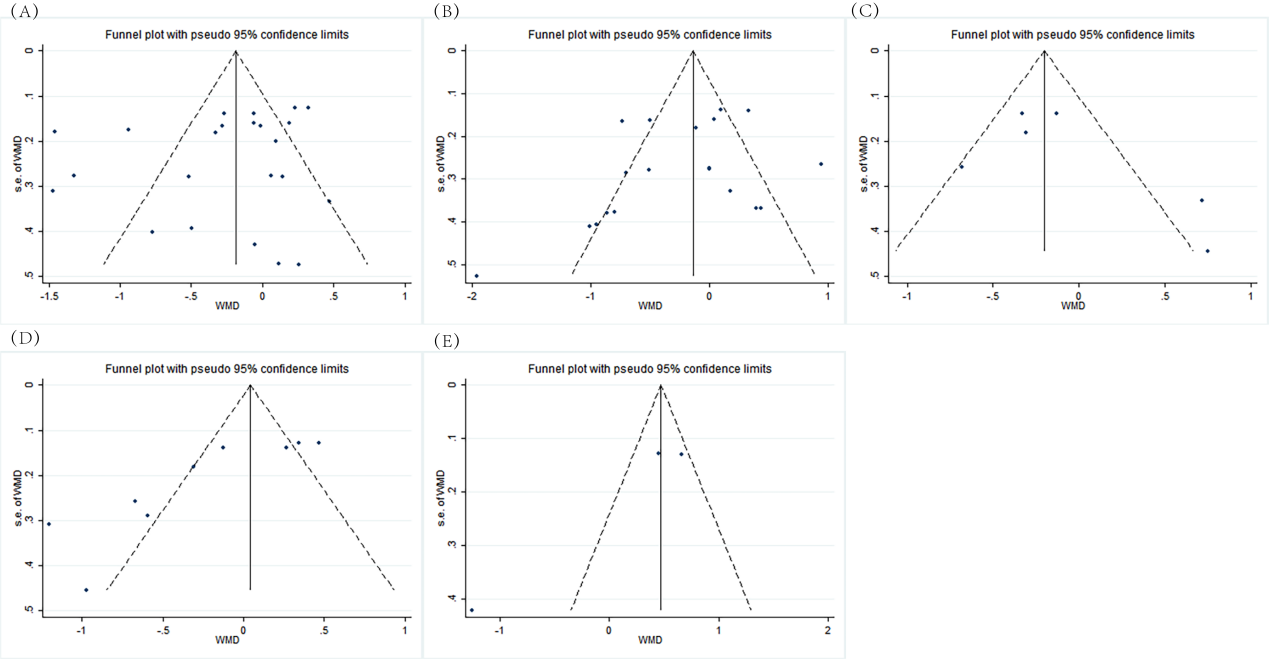


**Supplementary Figure 7.** Funnel plots of α-diversity: (A) Shannon index; (B) Simpson index; (C) ACE;(D)Chao1;(E)InvSimpson.


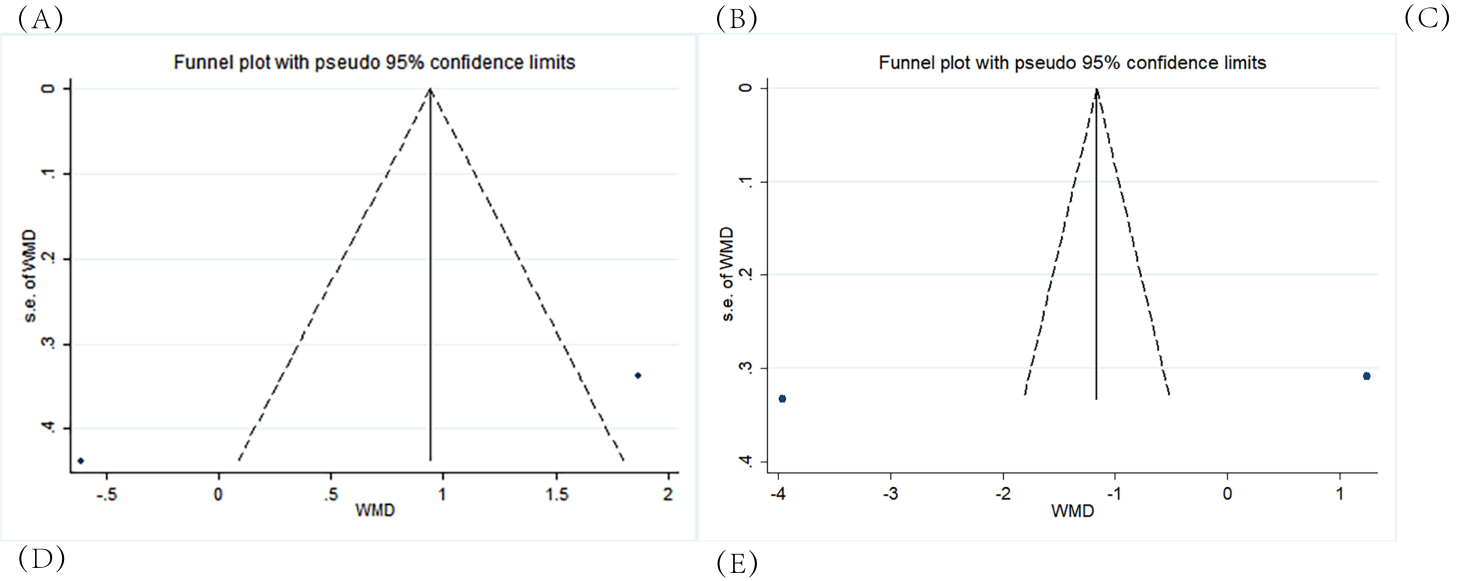


**Supplementary Figure 8.** Funnel plots of gut microbiota:(A)*Bacteroidetes* (B) *Bifidobacterium*.

*
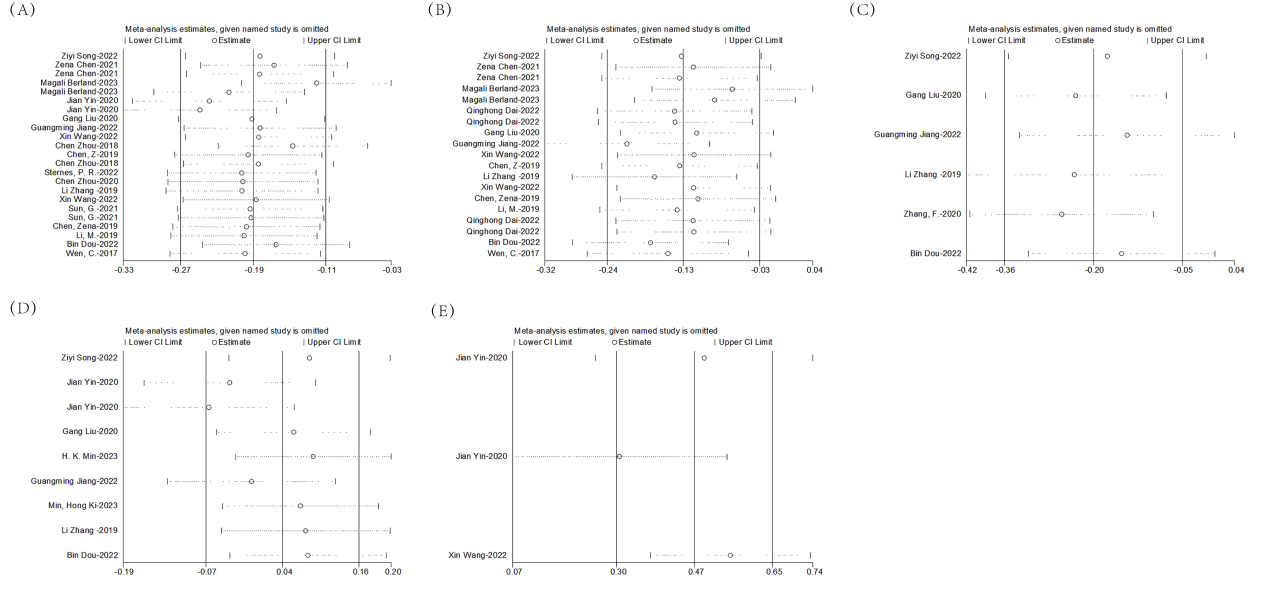
*

**Supplementary Figure 9.** Sensitivity analysis of α-diversity: (A) Shannon index; (B) Simpson index; (C) ACE; (D) Chao1; (E) InvSimpson.


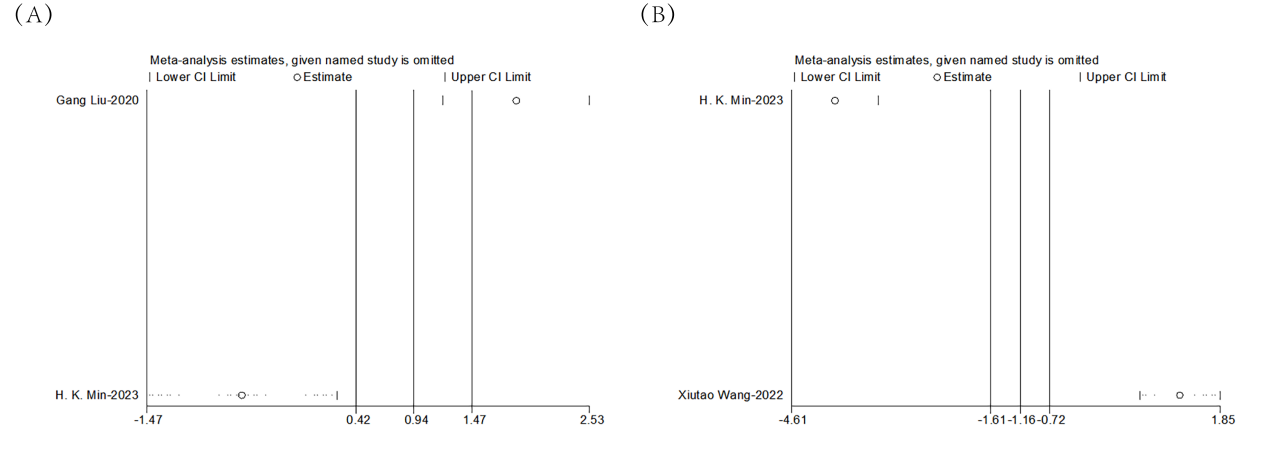


**Supplementary Figure 10.** Sensitivity analysis of gut microbiota:(A)*Bacteroidetes* (B) *Bifidobacterium*.
